# Comparison of Aqueous and Vitreous Lymphocyte Populations From Two Rat Models of Experimental Uveitis

**DOI:** 10.1167/iovs.18-24192

**Published:** 2018-05

**Authors:** Kathryn L. Pepple, Leslie Wilson, Russell N. Van Gelder

**Affiliations:** 1 Department of Ophthalmology, University of Washington, Seattle, Washington, United States; 2 Department of Biological Structure, University of Washington, Seattle, Washington, United States; 3 Department of Pathology, University of Washington, Seattle, Washington, United States

**Keywords:** experimental autoimmune uveitis, flow cytometry, intraocular lymphocyte, B-cell, T-cell

## Abstract

**Purpose:**

To compare lymphocyte populations present within inflamed eyes in two rat models of autoimmune uveitis.

**Methods:**

Experimental autoimmune uveitis (EAU) and primed mycobacterial uveitis (PMU) were initiated in Lewis rats. Aqueous and vitreous were collected at peak inflammation (PMU at day 2, EAU at day 14). The number of cells in the aqueous and vitreous was determined and compared for each eye and between the two models. Intraocular CD-19^+^ B cells, CD3^+^ T cells, and CD4^+^ or CD8^+^ T-cell subpopulations were identified by flow cytometry and compared between EAU and PMU.

**Results:**

The median number of cells/mL collected from PMU aqueous (7.98 × 10^7^ cells/mL), was not significantly different from the number of cells collected from EAU aqueous (1.61 × 10^7^ cells/mL, *P* = 0.94). EAU aqueous contains a significantly larger mononuclear population (median 61%, interquartile range [IQR] 44%–67%) than PMU (median 9%, IQR 8%–10% [*P* < 0.0001]). Within the mononuclear population, EAU and PMU aqueous demonstrate similar proportions of CD3^+^, CD4^+^ T cells. However, EAU has a larger CD3^+^, CD8^+^, T-cell population than PMU, and this population also demonstrates co-expression of CD45R. B cells comprise a significantly larger median percentage of cells in EAU aqueous (median 18%, IQR 15%–20%) compared to PMU (median 13%, IQR 9%–15%, *P* = 0.006).

**Conclusions:**

Flow cytometry analysis of intraocular lymphocytes from EAU and PMU identifies similarities and differences between the T-cell and B-cell populations present at peak inflammation. Complementary animal models that have well-defined mechanistic differences will improve our ability to test potential new therapies and bring meaningful advances into clinical practice for patients with uveitis.

Uveitis carries a high risk of vision loss and is estimated to cause 10% to 15% of blindness in the United States.^1–3^ Animal models that recapitulate a subset of uveitic conditions have been central to understanding the pathogenesis of disease.^4,5^ However, no single rodent model recapitulates all forms of human uveitis. Experimental autoimmune uveitis (EAU) is the most extensively studied model, and is the primary system from which the known mechanisms of ocular inflammation have been elucidated. However, this model assumes a purely autoimmune pathogenesis of disease. We have adapted a mixed innate and adaptive model of uveitis, previously utilized in larger mammals,^6,7^ as an alternative rodent model of uveitis, and named this primed mycobacterial uveitis (PMU).^8^ The two models are distinct as demonstrated by histologic, cytokine,^8^ and proteomic^9^ analysis. In PMU, uveitis is generated in a two-step process. First mycobacterial antigen is introduced to the immune system by subcutaneous injection. Then 7 days later, mycobacterial antigen is introduced directly into the eye by intravitreal injection. In this way, PMU models uveitis that develops after systemic exposure to an exogenous pathogen or antigen. By using a mycobacterial extract as the exogenous antigen, PMU is particularly well positioned to model two important and frequent forms of uveitis, tuberculosis (TB) and sarcoid associated uveitis.^10^ However, as toxoplasmosis and herpetic uveitis develop after prior systemic infection, PMU also has the potential to provide important insights into the mechanisms of these forms of uveitis.

The mechanisms of inflammation induction between EAU and PMU would be expected to generate a different range of effector cells infiltrating the eye during peak inflammation. As a model of autoimmune uveitis, EAU is dependent on T cells^11^ and generates an intraocular infiltrate that is T-cell and macrophage predominant.^12–16^ As a mixed model of inflammation, PMU eyes shows CD68^+^ macrophages as the predominant cell type on histologic sections, with a relatively small T-cell (CD3^+^) infiltrate.^8^ However, these analyses were histochemical and did not utilize flow cytometric analysis of live cells to quantitate the relative proportions of these populations. In particular, the role of B cells in ocular inflammation has not been explored as extensively as the other cell types, but with the success of anti-CD-20 therapies in some recalcitrant forms of human uveitis, a reconsideration of the B-cell compartment in experimental models has suggest there may be important roles for these cells in disease pathogenesis.^17^

In this study, we characterize differences in the lymphocyte populations of ocular infiltrating immune cells in PMU and EAU eyes utilizing flow cytometry. We also examined whether there are differences in the distribution of inflammatory subpopulations if the sample is obtained from the anterior chamber or the vitreous of the same eye.

## Methods

### Animals and Uveitis Induction

Female Lewis rats (*n* = 9) were purchased from Envigo (Cambridgeshire, UK) and maintained with standard chow and water ad libitum under specific pathogen-free conditions. The animal study protocol was approved by the Animal Care and Use Committee of the University of Washington (animal study protocol #4184-04) and was compliant with the ARVO Statement for the Use of Animals in Ophthalmic and Vision Research. PMU was generated as previously described.^8^ Briefly, animals received subcutaneous injection of 100 μg killed mycobacterium TB H37Ra antigen (#231141; Difco Laboratories, Detroit, MI, USA) in 0.1 cc of an emulsion of incomplete Freund's Adjuvant split into two equal doses to either hip (#263910; Difco Laboratories). Seven days later (designated as day 0), the right eye of each animal received an intravitreal injection of 5 μg of a suspension of killed mycobacterium TB H37Ra antigen in 5 μL phosphate-buffered saline (PBS). EAU was generated as previously described with subcutaneous injection of 30 μg interphotoreceptor retinoid binding protein peptide R16 (ADGSSWEGVGVVPDV) (Peptide 2.0, Chantilly, VA, USA) in 0.1 cc complete Freund's Adjuvant (2.5 mg/mL H37Ra in incomplete Freund's Adjuvant) in two divided doses to each hip on day 0.^18^ Clinical scoring was performed for both PMU and EAU animals using the previously reported score system for EAU.^18^ Briefly, 0 indicates no inflammation, 0.5 for dilated iris vessels, 1 for engorged blood vessels and pupillary contraction, 2 for hazy anterior chamber (AC) and decreased red reflex, 3 for moderately opaque AC but visible pupil and dull red reflex, and 4 for opaque AC and obscured pupil and absent red reflex.

### Optical Coherence Tomography (OCT) System, Image Acquisition, and Analysis

Anterior segment OCT images were acquired using the Bioptigen Envisu R2300 with the Bioptigen 18 mm telecentric lens (product #90-BORE-G3-18, Bioptigen, Inc., Morrisville, NC, USA). A 6 × 6 mm area was scanned with a density of 1000 A-scan/B-scan × 400 B-scans per anterior chamber volume. Anesthesia was provided with 68.2 mg/kg ketamine and 4.4 mg/kg xylazine IP (ketamine: Ketaset 100 mg/mL; Zoeitis, Inc., Kalamazoo, MI, USA; xylazine: AnaSed 20 mg/mL; Lloyd Laboratories, Shenandoah, IA, USA). Eyes were dilated with phenylephrine (2.5%, Akorn, Inc., Lake Forest, IL, USA) and corneal protection provided by drops of balanced salt solution (BSS) or Genteal gel (Alcon Laboratories, Inc., Fort Worth, TX, USA). Animals were wrapped in warming gauze and placed in the prone position in the Bioptigen rat imaging cassette. Images were obtained on day 7 (baseline) and day 2 (peak inflammation) for PMU animals, and on day 0 (baseline) and day 14 (peak inflammation) for EAU animals. A masked grader scored OCT images for the presence or absence of inflammation on the day of peak inflammation.^19^ Presence of inflammation included anterior chamber cell, hypopyon, pupillary membrane, and corneal edema.

### Aqueous and Vitreous Collection and Cell Counting

After imaging on the day of peak inflammation, animals were euthanized and samples collected for flow analysis. Prior to collection, each eye was washed with 1× PBS and dried with a Kimwipe (Kimberly-Clark Professional, Roswell, GA, USA). Corneal paracentesis was performed using a 30-gauge needle (Becton, Dickinson and Company, Franklin Lakes, NJ, USA), and aqueous humor collected from the ocular surface using a capillary tube (Sarstedt, Nümbrecht, Germany). Aqueous was transferred to an Eppendorf tube containing 90 μL cell collection buffer (1× PBS, 1× protease inhibitor [Sigma-Aldrich Corp., St. Louis, MO, USA], and 0.1% bovine serum albumin [BSA] [Fisher Scientific, Waltham, MA, USA]). Approximately 10 to 15 μL aqueous was collected from each eye. The eye was then enucleated and placed in a petri dish (Fisher Scientific). The cornea was removed at the limbus using Vannas scissors (World Precision Instruments, Sarasota, FL, USA), and the lens and vitreous removed together from the remaining eye cup. The vitreous (approximately 20 μL) was manually separated from the posterior lens and placed in a tube containing 90 μL cell collection buffer. Samples were stored on ice after collection and prior to staining. All microsurgical procedures were performed using a Leica M60 stereomicroscope (Leica Biosystems, Inc., Buffalo Grove, IL, USA) with coaxial illumination Leica KL1600 LED (Leica Biosystems, Inc.). Cell counting was performed on a Nexelcom Cellometer Auto 2000 Cell Viability counter (Nexelcom Bioscience, Lawrence, MA, USA) with acridine orange/propidium iodide (AO/PI) staining solution for live/dead mammalian nucleated cells (Nexelcom Bioscience). Cell number is reported as live cells/mL. No chemical or mechanical tissue disruption of the samples was performed prior to flow analysis.

### Flow Cytometry Sample Preparation, Data Acquisition, and Analysis

Samples were transferred to a 96-well Nunc conical well plate (Sigma-Aldrich Corp.) and spun at 450*g* at 4°C for 5 minutes. Cells were resuspended in 100 μL flow buffer (1× PBS with 1% BSA) with 5 ng/μL FC block (purified anti-CD32 clone D34-485, BD Biosciences, Reading, UK) and incubated at 4°C for 15 minutes. Staining was performed in the dark for 20 minutes at 4°C in flow buffer. The list of titrated surface markers for these experiments includes: CD3 clone 1F4 FITC (5 ng/μL), CD45R clone HIS24^20^ PE (4 ng/μL), CD8 clone OX-8 PerCP (2 ng/μL), and CD4 clone OX-35 PE-Cy7 (2 ng/μL). Live-dead discrimination was performed using FVS780 in the APC-Cy7 channel. All stains including the FVS780 dye were obtained from BD Biosciences. Unstained cell controls were used for gaiting analyses to distinguish positively from negatively staining cell populations. Compensation was performed using single color controls prepared from BD Comp Beads (BD Biosciences, Franklin Lakes, NJ, USA). Flow cytometric analysis was carried out with a FACS Canto II equipped with 488 and 647 nm excitation lasers. Data analysis was performed using FlowJo v10.1 software (FlowJo LLC, Ashland, OR, USA). Data presented represents results from three independent experiments. Population percentages and statistical results are presented for samples where >10,000 events were captured in the inflammatory gate.

### Statistical Analysis

Data were plotted and analyzed for statistical significance using Prism 7 GraphPad software (GraphPad Software, Inc., San Diego, CA, USA). Bonferroni correction was performed for multiple comparisons; calculated *P* values are presented. Comparison between cell number in the aqueous and vitreous was performed by Kruskal-Wallis test. Individual pairwise comparisons were tested using the Mann-Whitney *U* test. A value of *P* ≤ 0.01 was considered significant. Spearman correlation was used to test the association between cell number in aqueous and vitreous from individual eyes. Comparison of CD3^+^, HIS24^+^, (CD3^+^, CD4^+^) and (CD3^+^, CD8^+^) cell populations between EAU and PMU were performed using a Kruskal-Wallis test (*P* ≤ 0.01 considered significant). Pairwise comparisons between individual samples were performed using the Mann-Whitney *U* test (*P* ≤ 0.006 was considered significant given multiple comparisons).

## Results

### Quantification of Anterior Chamber and Vitreous Inflammatory Cells

**Figure 1:**
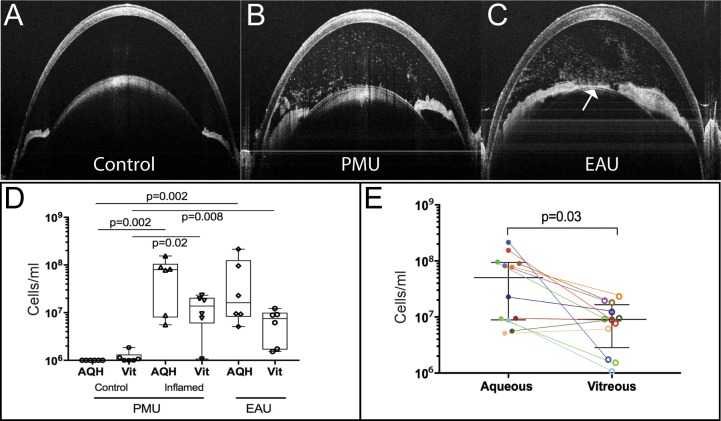
Aqueous and vitreous cell collection from individual eyes with experimental uveitis. Anterior chamber OCT images from (A) an uninflamed control eye with a clinical score of 0, (B) a PMU eye with a clinical score of 2, and (C) an EAU eye with a clinical score of 3. Inflammatory cells are seen as bright spots within the anterior chamber of the inflamed eyes (B, C). A pupillary membrane is also seen in C (white arrow). (D) Cell concentrations from the aqueous and vitreous of control, PMU, and EAU eyes. Box and whisker plots identify the median and the inner quartile ranges. The P values of the difference between samples are indicated. Kruskal-Wallis test on all samples (P = 0.0001). Individual pairwise comparisons were tested using the Mann-Whitney U test. (E) Cell concentration of the aqueous (filled circles) and vitreous (open circles) samples from inflamed eyes. Colored lines link samples from the same eye. The bars indicate the median and the IQR. Difference in cell concentration was significant at P = 0.03 by Mann-Whitney U test.

Uveitis induction was verified using clinical scoring and anterior chamber OCT in all animals prior to collection for flow analysis. Control eyes did not demonstrated inflammation prior to uveitis induction or on the day of sample collection (control eye clinical score = 0). All EAU and PMU eyes demonstrated anterior chamber inflammation by OCT on the day of peak inflammation (Figs. 1A–C). The average clinical EAU score on day 14 was 3 (range, 2–4). Average PMU clinical score on day 2 was 3 (range, 1–4). Aqueous and vitreous from each eye were collected separately, and the number of live cells/mL was determined from each chamber (Fig. 1D). The median number of cells/mL in the uninflamed control aqueous (6.9 × 10^4^ cells/mL) was significantly lower than in the inflamed PMU aqueous (7.98 × 10^7^ cells/mL, *P* = 0.002) and in the EAU aqueous (1.61 × 10^7^ cells/mL, *P* = 0.002). There was no difference between the number of cells in the inflamed PMU and EAU aqueous (*P* = 0.94). The median number of cells/mL in the control vitreous (7.43 × 10^5^ cells/mL) was significantly lower than in the PMU vitreous (1.37 × 10^7^ cells/mL, *P* = 0.01) and in the EAU vitreous (7.52 × 10^6^ cells/mL, *P* = 0.009). There was no significant difference between the number of cells/mL in PMU and EAU vitreous (*P* = 0.24).

To determine if there was a difference in the number of cells/mL recovered from the aqueous and vitreous in an inflamed eye, the number of cells/mL collected from the aqueous of all inflamed eyes (median 5.0 × 10^7^ cells/mL) was compared with the number of cells/mL collected from the vitreous (median 9.0 × 10^6^ cells/mL). While more cells were collected from the anterior chamber than from the vitreous, this difference was of borderline significance (*P* = 0.03) and likely due to incomplete cell recovery from the vitreous in the absence of chemical tissue digestion. To determine if the relative degree of inflammation was the same in both the aqueous and vitreous for each eye, the correlation between number of cells in the aqueous and vitreous from each eye was determined. There was no correlation between the number of cells in the aqueous and vitreous from individual inflamed eyes (*r* = 0.22, 95% confidence interval −0.42 to 0.71, *P* = 0.5). The relationship between the number of cells in the aqueous and vitreous of individual eyes is shown in Figure 1E.

### Quantification of Mononuclear and Granulocyte Populations in PMU and EAU

**Figure 2:**
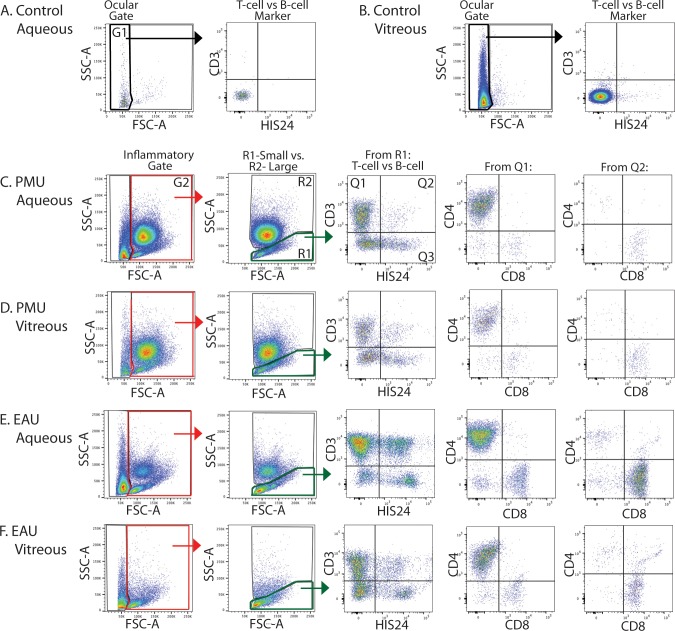
Different inflammatory cell populations are present in inflamed EAU and PMU eyes. Control eye (A) aqueous and (B) vitreous were used to identify the ocular cell gate (G1 bracketed in bold black in panels A and B) based on SSC and FSC (also see Supplementary Fig. S1). More than 98% of cells from this gate were negative for lymphocyte markers CD3 or HIS24. Cells within the inflammatory gate (G2 and bracketed in red in C–F) were analyzed for (C) PMU aqueous, (D) PMU vitreous, (E) EAU aqueous, and (F) EAU vitreous. Within the inflammatory gate, mononuclear cells were gated within R1 (bracketed in green) and granulocytes were quantified from within the R2 gate. Mononuclear in R1 were further analyzed for the expression of the T-cell marker CD3 and the B-cell marker HIS24 (CD45R). CD3^+^ populations from Q1 (CD3^+^, CD45R^−^) and Q2 (CD3^+^, CD45R^+^) were then analyzed for the expression CD4 or CD8. Q3 (CD3^−^, CD45R^+^) represents the B-cell population. Monocytes and macrophages are present in Q4 (CD3^−^, CD45^−^).

Control eyes were used to determine the gating strategy that would discriminate between native eye cells (ocular gate) and infiltrating inflammatory cells (inflammatory gate) based on side scatter (SSC) and forward scatter (FSC) characteristics after selection for live, single cells (full gating strategy in Supplementary Fig. S1). Plotting SSC against FSC identified that at least 99% of events were located below 70K on the FSC axis in an uninflamed control eye. This area was denoted as the ocular gate (black). The inflammatory gate (red) included the remaining area. In control eyes, <1% of positive staining events for inflammatory cell markers (CD3, HIS24, CD8, CD4) were detected from the ocular and inflammatory gates in the aqueous and vitreous (Figs. 2A, 2B).

**Figure 3:**
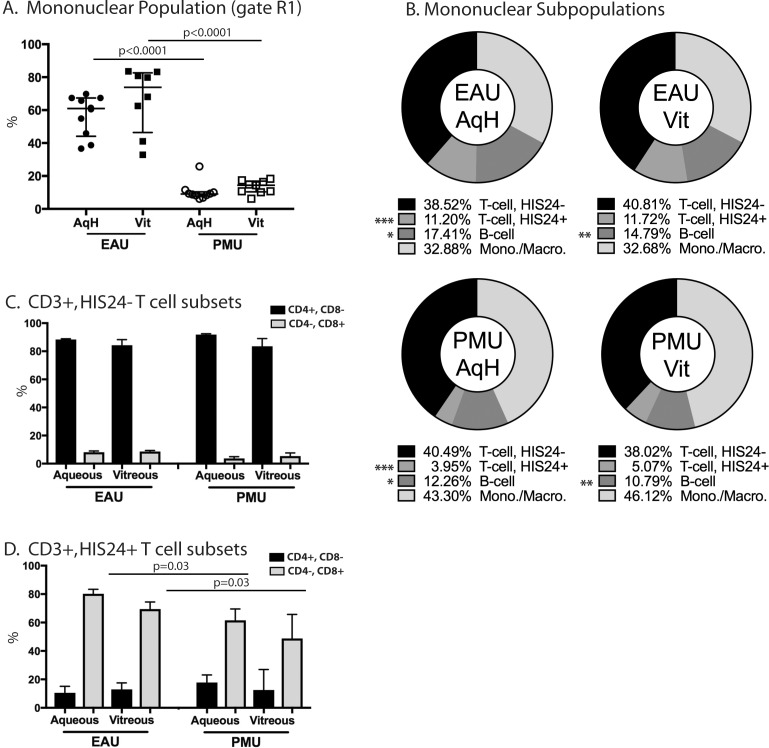
Significant differences are present in the ocular infiltrating leukocyte populations between EAU and PMU, but not between the aqueous and vitreous from the same model. (A) Graph demonstrating the percentage of inflammatory cells falling within the mononuclear R1 gate (green bracket in Fig. 2) from EAU and PMU aqueous (AqH) and Vitreous (Vit). The difference between these populations was significant (P < 0.0001). (B) From the R1 gate, expression patterns of the T-cell marker CD3 and B-cell marker HIS24 were determined. T cells include the CD3^+^, HIS24^−^ and CD3^+^, HIS24^+^ populations. B cells are CD3^−^, HIS24^+^. Monocytes and macrophages are CD3^−^, HIS24^−^. Population average % is shown for each quadrant for each sample. Pairwise comparisons between EAU and PMU samples from the same chamber were performed using the Mann-Whitney U test (P ≤ 0.006 was considered significant given multiple comparisons). Significant differences are indicated * = 0.006, ** = 0.003, *** <0.001. (C) CD3^+^, HIS24^−^ T cells predominantly express CD4, not CD8. (D) The majority of CD3^+^, HIS24^+^ T cells express CD8. Pairwise comparisons between individual samples were performed using the Mann-Whitney U test.

The predefined gating strategy was then applied to EAU and PMU eyes (full gating strategy examples Supplementary Figs. S2, S3). From PMU and EAU eyes, the cells in the inflammatory gate were divided into two subgroups based on their intrinsic light scattering properties (Figs. 2C–F). Region 1 (R1) encompasses the population containing mononuclear cells including lymphocytes. Region 2 (R2) encompasses a population cells with higher scatter consistent with granulocytes. The percentage of cells in the R1 (mononuclear) and R2 (granulocyte) from each chamber were calculated and compared between PMU and EAU eyes (Fig. 3A). EAU aqueous and vitreous were made up primarily of mononuclear cells. In the EAU aqueous, the mononuclear population median percentage was 61% (interquartile range [IQR] 44%–67%). In the EAU vitreous, the mononuclear population median percentage was 74% (IQR 46%–83%). In contrast, PMU eyes had a significantly lower percentage of cells in the mononuclear population with the aqueous median of 9% (IQR 8%–10%) (*P* < 0.0001) and vitreous median of 14% (IQR 10%–16%) (*P* < 0.0001). The R2 (granulocyte) population was the predominant population in PMU eyes. In the PMU aqueous, the granulocyte population median percentage was 91% (IQR 89%–92%). In the PMU vitreous, the granulocyte population median percentage was 84% (IQR 82%–89%). In contrast, EAU eyes had a significantly lower percentage of cells in the granulocyte population than PMU with the aqueous median of 39% (IQR 32%–56%) (*P* < 0.0001) and vitreous median of 23% (IQR 17%–53%) (*P* < 0.0001).

### Intraocular T-Cell and B-Cell Populations in EAU and PMU

From all EAU and PMU samples <1% of events from the R2 gate were positive for either CD3 or CD45R, and no additional analysis was performed on the R2 population (data not shown). From within the R1-mononuclear gate, the T-cell and B-cell populations were separated using the anti-CD3 clone 1F4 for T cells and the anti-CD45R clone HIS24 for B cells (Fig. 2). HIS24 recognizes a developmentally regulated form of CD45R found on most B cells, but not on plasma cell or cells of the myeloid or erythroid lineages in rats.^20^ Only cells that were CD3^−^, HIS24^+^ are considered B cells in this analysis. From the R1 gate, four distinct populations were identified based on their pattern of staining with CD3 and HIS24 (Fig. 3B). Shown in Q1 and Q2, roughly half of the small cells in EAU eyes were CD3^+^ (mean aqueous 49.7% ± 17.4%, vitreous 52.5% ± 10.5%). In PMU eyes, just under half of the small cells were CD3^+^ (mean aqueous 44.5% ± 13.6%, vitreous 43.1% ± 9.2%), but this was not significantly different from EAU eyes (*P* = 0.41). In addition, there was no difference in the size of the predominant CD3^+^ HIS24^−^ (Q1) populations (Supplementary Table S1). The second group of T cells is found in Q2, and is defined by CD3 and CD45R double positive staining. This population is present in both EAU and PMU eyes, but is significantly larger in EAU eyes than in PMU eyes in both the aqueous (EAU 11.20% ± 5.04% vs. PMU 3.95% ± 2.35%, *P* < 0.001) and the vitreous (EAU 11.72% ± 5.61% vs. PMU 5.07% ± 2.10, *P* = 0.003).

Due to the presence of the two populations of CD3^+^ cells based on the co-expression or absence of HIS24 staining, we wondered if there was a T-cell lineage specificity that could explain the unexpected expression of this B-cell CD45R isoform in a T-cell population. To address this question, we looked at the distribution of CD4^+^ and CD8^+^ within the HIS24^−^ (Q1) and HIS24^+^ (Q2) T cells in the aqueous (Figs. 2, 3C, 3D). Within the CD3^+^, HIS24^−^ population, CD4^+^, CD8^−^ T cells were the predominant cell type (>80%) for both EAU and PMU. In contrast, within the CD3^+^, HIS24^+^ population, the CD4^−^, CD8^+^ T cells were the predominant cell type in EAU aqueous (median 80%, IQR 72%–83%) compared to PMU aqueous (median 61%, IQR 39–69). This difference was significant (*P* = 0.03). The combined expression of CD3, CD8, and CD45R identified here suggests that the previously reported B-cell specific CD45R isoform identified by the HIS24 antibody is also expressed on a subset of T cells, predominantly within the CD8^+^ T-cell compartment in inflamed Lewis rat eyes.

In addition to the identification of T cells within the eyes of both EAU and PMU eyes, a B-cell population (CD3^−^, HIS24^+^ cells in Q3) was identified. This population comprises around 16% of the mononuclear cells in EAU and 12% of mononuclear cells in PMU eyes. While this population was slightly larger in EAU than in PMU eyes, the difference was only significant in the aqueous (EAU 17% vs. PMU 12%, *P* = 0.006) but not in the vitreous (EAU 15% vs. PMU 11%, *P* = 0.043). Finally, in addition to the positive staining populations, a CD3^−^, HIS24^−^ double negative population (Q4) was identified in both models, and likely represents the macrophage population. The size of this population was not significantly different between the models, ∼30% in EAU vs. ∼40% in PMU (*P* = 0.12).

## Discussion

We report here a comparative analysis of the lymphocyte populations from two models of experimental uveitis in Lewis rats. Both models produced robust panuveitis at peak inflammation. This is in contrast to EAU in the mouse, which produces primarily retinal inflammation.^18^ We found significant differences in the overall contribution of lymphocytes to each model, with more than 60% of the inflammatory cells in EAU falling within the monocytic R1 gate compared to less than 15% in PMU. Conversely, in PMU, the majority of inflammatory cells fell within the R2 gate and demonstrated higher SSC and FSC characteristics consistent with granulocytes.^21^ Within the R1 gate, the overall distribution of lymphocyte subtypes (B cell versus T cell) are quite similar between the models, despite differences in overall larger proportion of the lymphocytes in the EAU model. Another similarity between the models includes the predominance of the CD3^+^CD4^+^ T-cell subset at the peak of inflammation, a finding previously reported for EAU.^12,22–24^ Our results also suggests that despite the substantial innate response generated by the direct injection of an exogenous antigen directly into the vitreous in PMU, there is an important contribution of adaptive immunity to the PMU model, and one that looks remarkably similar to the response in EAU. The adaptive contribution to the PMU response is likely generated by the systemic “prime” of subcutaneous antigen that the animals experience 7 days prior to the intravitreal injection. Additional studies will be required to determine if there are differences in the T-cell response in unprimed animals, and to explore the antigen specificity of the response in PMU.

The HIS24 antibody recognizes a 205-kDa developmentally regulated isoform of CD45R expressed primarily in B cells prior to plasma cells differentiation.^20^ Previous studies have identified that cells of myeloid and erythroid lineage and the majority of thymocytes are HIS24 negative.^20^ However, the CD45R isoform recognized by HIS24 is not expressed uniquely on B cells, as these authors also reported rare HIS24-positive, IgG-negative cells in the medulla of the thymus and in the thoracic duct. In this study, we identified a CD3, HIS24 double positive population confirming that the CD45R isoform is co-expressed on some T cells during active ocular inflammation in the rat. Furthermore, we determined that this co-expression is predominantly found on CD8^+^ T cells. This is in contrast to a previous study that identified co-expression of the CD45RC isoform exclusively with CD4 but not CD8 T cells isolated from ocular draining lymph nodes in Lewis rats with EAU.^25^ In this study, Han et al.^25^ determined that while the CD4^+^, CD45RC^+^ T cells were uveitogenic, the CD8^+^, CD45RC^low^ T cells suppressed inflammation. Further functional characterization of the CD3^+^, CD8^+^, CD45R^+^ cells identified in our study could provide additional information about how different subtypes of T cells contribute to the pathobiology of ocular inflammation. Additionally, since the population of CD3^+^, CD45R^+^ T cells were a significantly larger part of the overall lymphocyte population in EAU than in PMU, understanding the role of these cells may provide important insight about mechanistic differences between the models.

The presence of a B-cell population within the inflamed eye has been reported previously in animal models of uveitis, and there has been a renewed interest in the role of B cells in noninfectious uveitis due to benefits in patients treated with the B-cell depleting therapy with rituximab (reviewed in Ref. 17). In this study, we identified a HIS24^+^, CD3^−^ population of infiltrating B cells in both models of uveitis. EAU had a larger B-cell population than PMU, which is consistent the primary role of the adaptive immune response in EAU. However, B cells still comprised over 10% of the small leukocytes in PMU, and identification of population was unexpected. In prior studies in EAU in mice and guinea pigs, when B cells were identified, they were a limited proportion of infiltrating cells (≤5%),^12,26^ more commonly identified in chronic lesions, areas of choroidal neovascularization, or at late stages of the disease.^27^ The exact role of intraocular B cells in uveitis is not well understood, and they may have different functions and importance in each of the uveitis models. However, the presence of this population in both experimental forms of uveitis supports further studies to determine what role B cells play in initiating or regulating ocular inflammation.

Finally, we did not find significant differences in the lymphocyte composition of the aqueous and vitreous of the same eye. Previous studies in experimental uveitis in mice have analyzed the inflammatory infiltrate recovered from excised retinas, whole eyes, or residual globes after lens removal rather than the isolated aqueous and vitreous.^13,22,23,28,29^ Our data show that in rats, the cellular populations between the two chambers are not significantly different. We did find that more cells were collected from the aqueous than the vitreous, were technically straightforward to collect, and generated a single-cell preparation without the need for mechanical or chemical digestion. This has important implications for studies in humans and larger animal models where the aqueous can be sampled safely and easily in vivo.^30,31^ Our data suggest immunophenotyping of anterior chamber cells could provide very similar information as vitreous sampling provides, but without requiring the more invasive procedure of a vitrectomy. In experimental models, future studies could be performed to extend this comparison to include the correlation between aqueous and intraretinal or choroidal inflammatory cell populations.

In summary, both EAU and PMU exhibit features that recapitulate a subset of the human disease phenotype, and together provide good coverage of the spectrum of disease seen in human patients. In addition to being a model for TB and sarcoid uveitis,^8,32^ PMU may also provide insight into mechanisms underlying other forms of uveitis that are not well represented by EAU, such as toxoplasmosis panuveitis or HLA-B27 acute anterior uveitis, which develop after a systemic exposure to the pathogen or antigen.^33^ Using complementary models that have well defined mechanistic differences to study disease pathobiology and to test potential therapies will help improve our ability to bring meaningful insights into clinical practice.

## Supplementary Material

Supplement 1
