# Supplementary material for: Comparison of Aqueous and Vitreous Lymphocyte Populations From Two Rat Models of Experimental Uveitis
Source: Invest Ophthalmol Vis Sci. 2018 May;59(6):2504–11. doi: 10.1167/iovs.18-24192 (PMC5963002; doi:10.1167/iovs.18-24192)
Supplement: Supplement 1 [file iovs-59-06-16_s01.pdf]

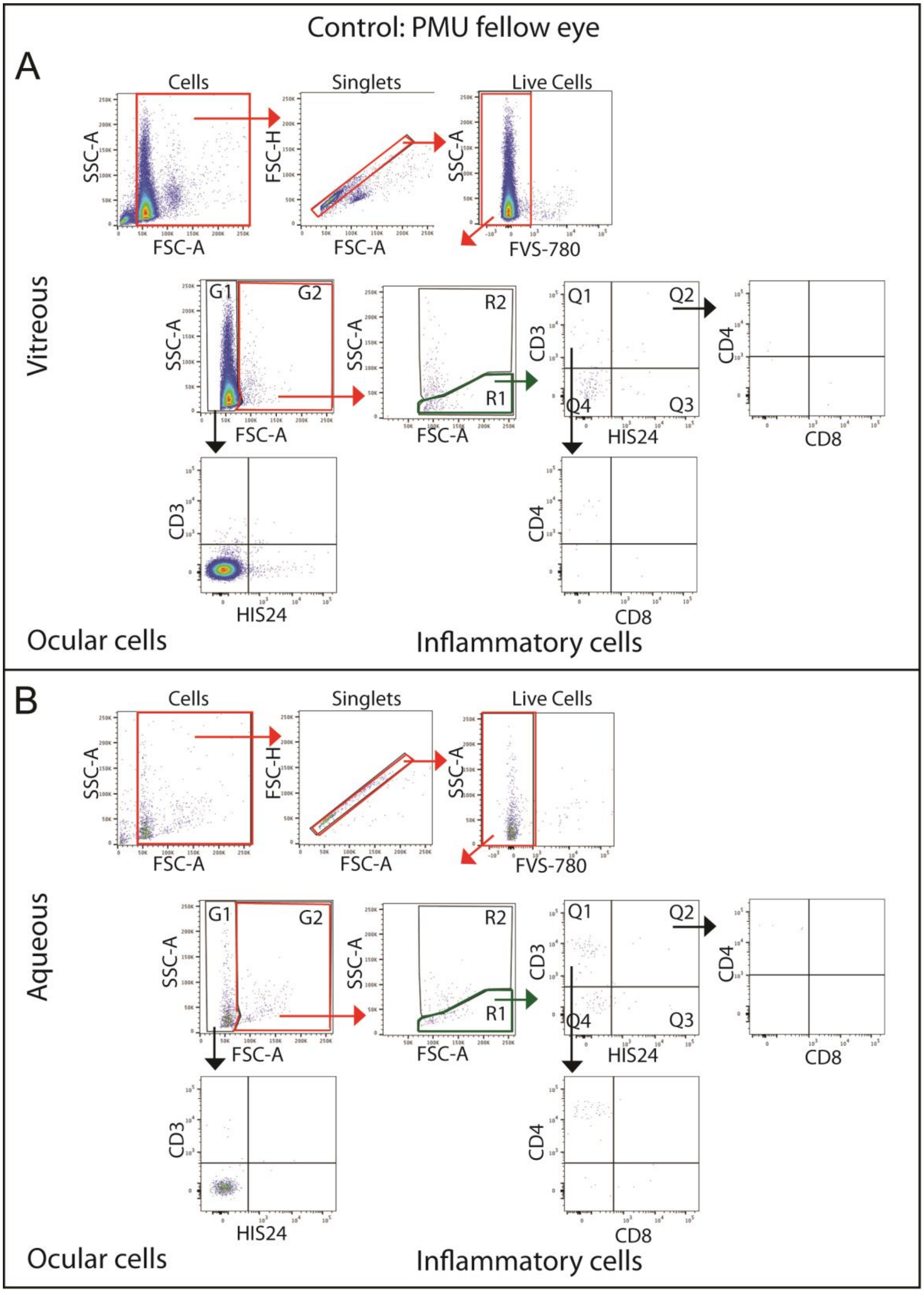

Supplemental Figure 1

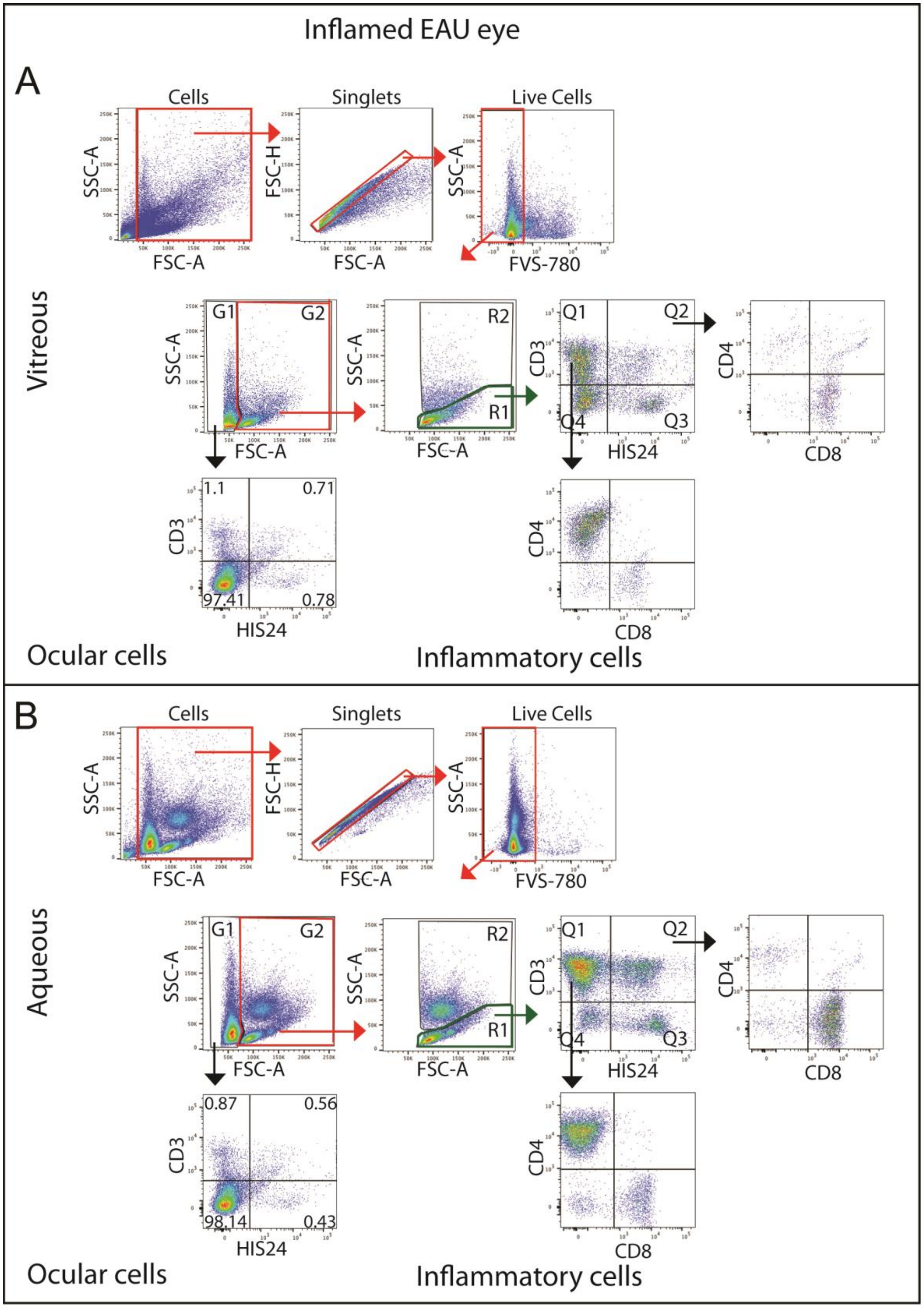

Supplemental Figure 2

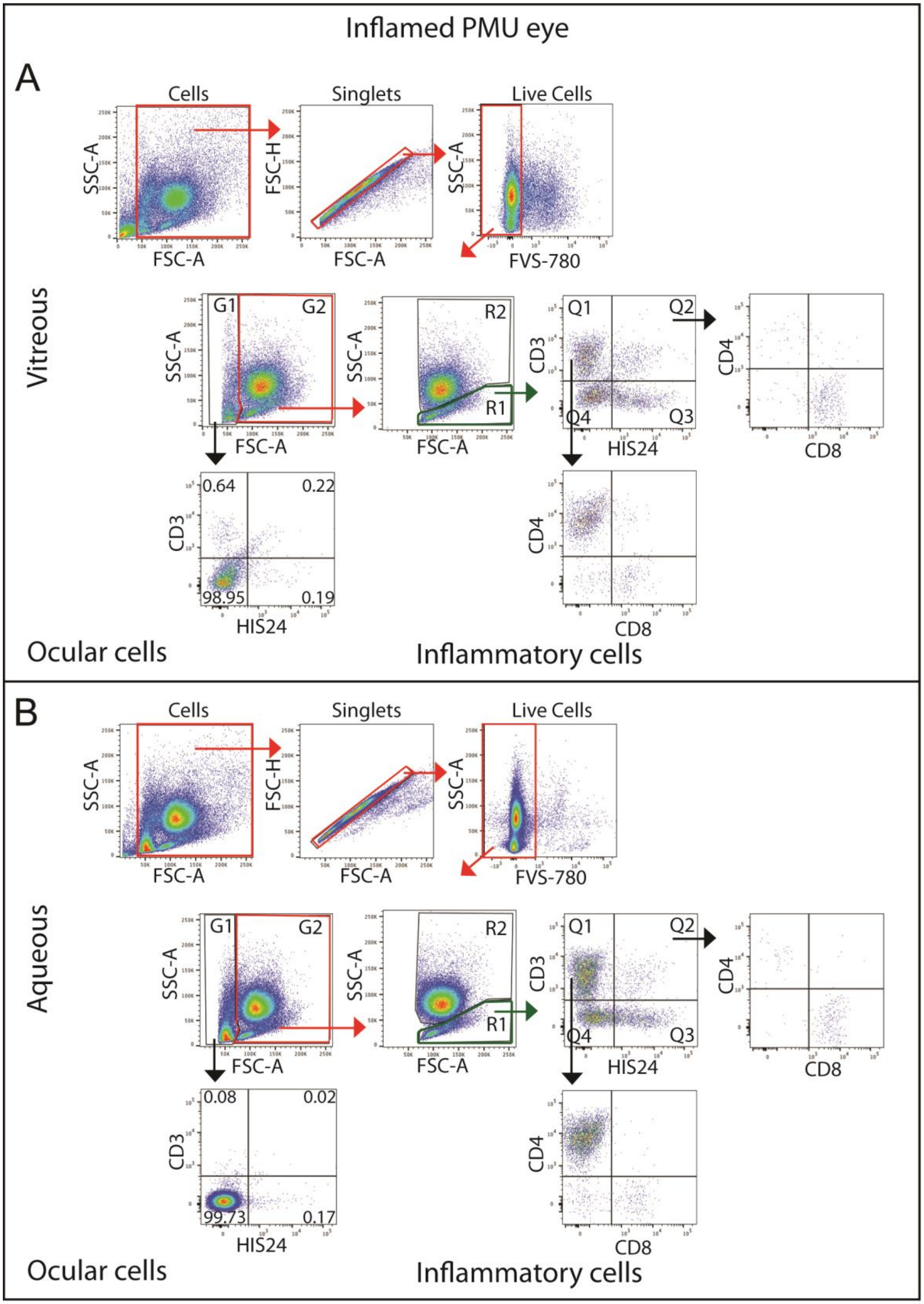

Supplemental Figure 3

Supplemental Figure 1. Determination of the ocular versus inflammatory cell flow cytometry gating strategy using uninflamed PMU control eyes. Analysis of the **A.** vitreous and **B.** aqueous compartments from a representative control eye. The strategy shows sequential exclusion of debris (cell gate), selection for single cells (singlets gate), and live dead discrimination (live cells gate) before splitting the SSC-A and FSC-A axis into the "ocular" gate (G1, bracketed in black) and the "inflammatory" gate (G2, bracketed in red). From the ocular gate (G1), the leukocyte surface markers for T-cells (CD3), B-Cells (HIS24) identified that >98% of events were negative for both cell markers. From the inflammatory gate (G2) cells were further divided into the mononuclear population (gate R1, bracketed in green), and the granulocyte population (gate R2). The population in R1 was then separated based on expression patterns of CD3 and HIS24 into 4 quadrants (Q1-Q4). The CD3+ populations in quadrants Q1 and Q2 were then separated based on the expression of CD4 and CD8. In control eyes insufficient cells (<10,000) were present in the inflammatory gate (G2) for statistical analysis.

Supplemental Figure 2. Full gating strategy for an EAU eye. Analysis of the **A.** vitreous and **B.** aqueous compartments from a representative EAU eye. Percentage of cells in the ocular gate expressing CD3 and CD45R are shown and total < 3% of ocular gate cells in the vitreous and <2% of ocular gate in the aqueous.

Supplemental Figure 3. Full gating strategy for a PMU eye. Analysis of the **A.** vitreous and **B.** aqueous compartments from a representative PMU eye. Percentage of cells in the ocular gate expressing CD3 and CD45R are shown and total < 2% of ocular gate cells in the vitreous and <1% of ocular gate in the aqueous.

	EAU AqH % ± SD	PMU AqH % ± SD	p-value	EAU Vit % ± SD	PMU Vit % ± SD	p-value
**T-cell, HIS24-**	38.52 ± 13.01	40.50 ± 12.02	0.58	40.81 ± 6.82	38.02 ± 8.89	0.54
**T-cell, HIS24+**	11.20 ± 5.04	3.95 ± 2.35	<0.001	11.72 ± 5.61	5.07 ± 2.10	0.003
**B-cell**	17.41 ± 3.30	12.26 ± 3.71	0.006	14.79 ± 4.00	10.79 ± 4.52	0.04
**Mono./ Macrophage**	32.88 ± 15.01	43.31 ± 16.02	0.36	32.68 ± 11.81	46.12 ± 11.91	0.03

Supplemental Table 1. Mononuclear subpopulations in the R1 gate

Mononuclear subpopulations in the R1 qate were separated by expression of CD3 and HIS24, and the populations from EAU and PMU eyes were compared. Average % of the R1 population ± the standard deviation (SD) in each chamber is shown. Pairwise comparisons between individual samples were performed using the Mann-Whitney test ($p \leq 0.006$ was considered significant given multiple comparisons). Experimental autoimmune uveitis (EAU), primed mycobacterial uveitis (PMU), aqueous humor (AqH), vitreous (Vit), standard deviation (SD), monocyte (mono.).
